# To Cut the Mustard: Antimicrobial Activity of Selenocyanates on the Plate and in the Gas Phase

**DOI:** 10.3390/antibiotics12020290

**Published:** 2023-02-01

**Authors:** Muhammad Sarfraz, Muhammad Jawad Nasim, Martin C. H. Gruhlke, Jadwiga Handzlik, Claus Jacob

**Affiliations:** 1 Department of Plant Physiology, RWTH Aachen University, Worringer Weg 1, 52056 Aachen, Germany; 2 Division of Bioorganic Chemistry, Saarland University, Campus B2 1, 66123 Saarbruecken, Germany; 3 Department of Technology and Biotechnology of Drugs, Faculty of Pharmacy, Jagiellonian University Medical College in Kraków, Medyczna 9, 30-688 Cracow, Poland

**Keywords:** gas phase activity, (iso-)thiocyanates, microbiological assays, selenocyanates

## Abstract

Organic selenocyanates (RSeCN) are among the most reactive and biologically active Se species, often exhibiting a pronounced cytotoxic activity against mammalian cells and microorganisms. Various aromatic selenocyanates have been synthesized and, similar to some of the most Reactive Sulfur Species (RSS), such as allicin, found to be active against a range of bacteria, including *Escherichia coli*, *Pseudomonas syringae* and *Micrococcus luteus*, and fungi, including *Verticillium dahlia*, *Verticillium longisporum*, *Alternaria brassicicola*, and *Botrytis cinerea*, even via the gas phase. The highest antimicrobial activity has been observed for benzyl selenocyanate, which inhibited the growth of all bacteria considerably, even at the lowest tested concentration of 50 µM. Notably, neither the analogues thiocyanate (BTC) nor isothiocyanate (BITC) show any of these activities, rendering this selenium motif rather special in activity and mode of action. Eventually, these findings advocate a range of potential applications of organic selenocyanates in medicine and agriculture.

## 1. Introduction

Organic selenocyanates (RSeCN) form an interesting class of compounds with a pronounced chemical reactivity and also extraordinary, often toxic, biological activities [1]. Unlike other Reactive Selenium Species (RSeS), such as selenols, diselenides and, more recently, seleninic acids, selenocyanates have often been ignored by biological chemists who have placed them in the drawer of potent precursors than potent antioxidants or antibiotics [2,3,4]. Perhaps the often unfounded notion that such selenocyanates readily release cyanide (CN^−^) ions may have hampered their wider application in biology.

**Figure 1:**
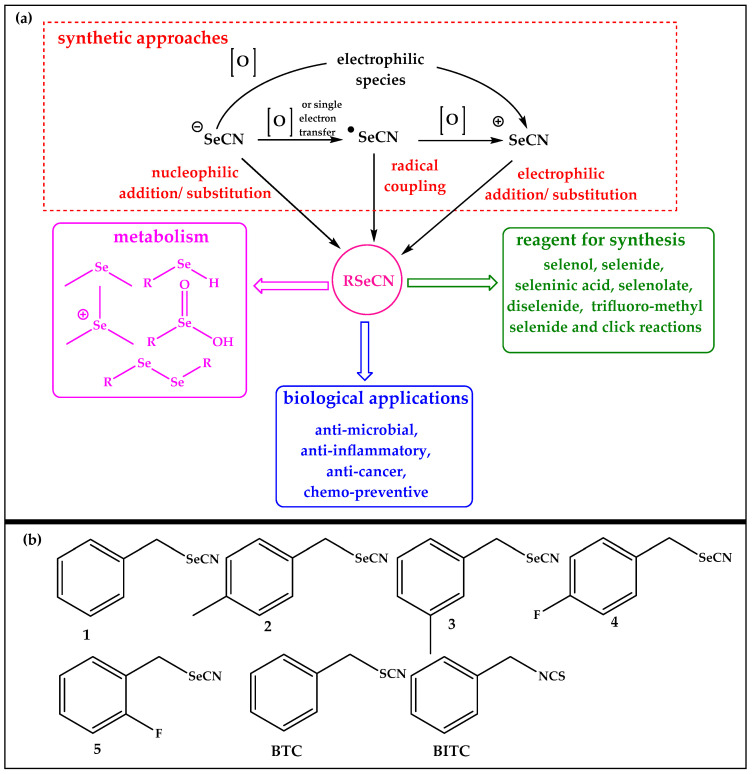
Panel (**a**) represents various approaches to synthesize RSeCN, which include not only electrophilic or nucleophilic addition and substitution reactions but also radical coupling reactions. Once produced, these multifunctional agents may be exploited for the generation of other RSeS. The metabolism of selenocyanates also leads to the formation of seleninic acid and diselenide, along with dimethyl selenide and trimethyl selenonium ions. RSeCN provide several important biological applications. Panel (**b**) represents the structures of compounds employed in this study.

From the perspective of reactivity, this is rather unfortunate, as organic selenocyanates combine elements of nucleophilic and electrophilic reactivity, are prone to substitution, hydrolysis, redox changes and metabolism, and, as we shall see, may even exert their activities contact-less, from a distance, via the gas phase [1,5,6,7]. Organic selenocyanates can also be synthesized by a variety of different routes, as described in the literature and presented in Figure 1, Panel a [1]. Selenocyanates have been reported to exhibit excellent anti-leishmanial, antichagasic, anticancer and antibacterial activities [8,9,10]. Moreover, members of this class of RSeS are able to modulate oxidative stress, attenuate catalase activity, and upregulate superoxide dismutase in cultured neuronal cells exposed to H_2_O_2_ [11]. Selenocyanates inhibit lung tumors in mice and prostate cancers in humans [12,13]. In order to exploit the possible biological activities and applications in more detail, we have recently synthesized a series of organic selenocyanates (Figure 1, Panel b). These compounds have already shown some promising cytotoxic activity in human cancer cell lines, albeit with limited selectivity [14].

Inspired by the fact that simple organic (iso)thiocyanates, such as allyl isothiocyanate (C_3_H_4_NSC), from the famous mustard oil, are among the most potent natural antimicrobial agents, we have also tested these selenocyanates against a range of bacteria and fungi [15,16,17,18,19,20]. Since the allyl derivatives are generally extremely unstable, we synthesized a series of rather stable, solid (at room temperature) in nature benzyl selenocyanates. In previous studies, we have reported the efficacy of these compounds against highly resistant ESKAPE bacteria [14]. Moreover, yeast-based chemogenetic screening has revealed that these compounds may attack the glutathione metabolism and the glutathione pool in the target cells [21]. Here, we report a wide range of activities against bacteria and fungi, which is specific for selenocyanates, rather than thiocyanates, and can also be administered via the gas phase.

## 2. Materials and Methods

### 2.1. Selenocyanates and (Iso)Thiocyantes

Aliphatic selenocyanates and isoselenocyanates, including the selenium analogue of mustard oil, are notoriously unstable chemically and often sensitive towards water and air [22]. In contrast, aromatic selenocyanates, such as phenyl- and benzyl-selenocyanates, are more stable and amenable to experiments under physiological conditions. Selenocyanates (1–5) shown in Figure 1 have, therefore, been selected for this study and have been synthesized as described by us in a previous publication [14]. The sulfur analogues benzyl thiocyanate (BTC) and benzyl isothiocyanate (BITC) have been employed for comparison (Figure 1).

### 2.2. Organisms and Media

Bacteria such as *Escherichia coli K12* and *Pseudomonas syringae* 4612 were employed as representative Gram-negative bacteria and *Micrococcus luteus* as a representative Gram-positive bacterium. *E. coli* and *M. luteus* were cultured at 37 °C and 28 °C, respectively, on Luria-Bertani broth (LB) medium (10 g L^−1^ tryptone, 5 g L^−1^ yeast extract, 5 g L^−1^ NaCl). 15 g L^−1^ of agar was added for solid medium, followed by sterilization via autoclave. *P. syringae* was cultured at 28 °C by using King’s B medium (20 g L^−1^ peptone, 12.6 g L^−1^ glycerol, 1.5 g K_2_HPO_4_ (anhydrous)) and 1.5 g L^−1^ MgSO_4_ (heptahydrate) was added after sterilization. 15 g L^−1^ of agar were added for solid medium to culture *P. syringae*. Chemicals were purchased from Carl Roth, Karlsruhe, Germany [23,24,25] 

Plant pathogenic fungi, including *Verticillium dahlia*, *Verticillium longisporum*, *Alternaria brassicicola* and *Botrytis cinerea*, were cultured in Potato Dextrose Broth (PDB) as liquid growth medium and on Potato Dextrose Agar (PDA) as solid medium. Both PDB and PDA were purchased from Merck, Darmstadt, Germany.

### 2.3. Assessment of Antibacterial Activity

The efficacy of compounds against *E. coli*, *P. syringae*, and *M. luteus* was evaluated following the routine microbial assays based on optical density (OD) and measured in the form of growth curves. The antibacterial activities of compounds against *M. luteus, E. coli*, and *P. syringae* were performed according to the protocols already described in literature [23,24,25]. Bacterial culture with growth medium was employed as negative control. Bacterial cell densities were determined at the wavelength of 600 nm (OD_600_) using a Tristar2 LB 942 Multimode Reader (Berthold Technologies GmbH & Co. KG, Bad Wildbad, Germany). The compounds were tested at final concentrations of 50, 100, 150 and 200 μM.

### 2.4. Drop Test

The drop test was performed according to the protocols described in the literature. Control plates contained only growth medium with drops of bacterial culture. The plates were incubated for 48 h at 37 °C for *E. coli* and 28 °C for *M. luteus* [21,26].

### 2.5. Inhibition Zone Assay

The antimicrobial activity of compounds in terms of zones of inhibitions was determined according to protocol described in the literature. Three different concentrations of compounds (2, 4 and 8 mM) were evaluated against *E. coli*, *P. syringae*, and *M. luteus*. The inhibition zone diameters were determined, and photographs of the plates were taken [27].

### 2.6. Determination of Antibacterial Activity of Compounds in the Gas Phase

The antibacterial activities of compounds in gas phase were determined employing the drop test with a few modifications. A drop of each compound (20 µL, 80 mM) was placed at the center of the lid, and the Petri plates containing bacterial culture mediums were inverted over the lid. The plates were subsequently incubated over night at a particular temperature (*M. luteus* and *P. syringae* at 28 °C, with *E. coli* at 37 °C) [26].

### 2.7. Evaluation of the Antifungal Activity

The antifungal activity of compounds was determined against plant pathogenic fungi, *V. dahliae*, *V. longisporum*, *A. brassicicola* and *B. cinerea*, employing the protocol described in the literature. The activity was determined at a concentration of 4 mM [28,29]. Plates were incubated for 1 week at a temperature of 22 °C, and then photographed.

### 2.8. Determination of Antifungal Activity of Compounds in the Gas Phase

The antifungal activities of compounds in the gas phase were determined employing the drop test with a few modifications. A drop of each compound (20 µL, 80 mM) was placed at the center of the lid, and the Petri plates containing fungal culture mediums were inverted over the lid. The plates were subsequently incubated at a temperature of 22 °C. The plates were photographed after 5 days of the treatment [26,29].

### 2.9. Calculation of Vapor Pressure

The values for calculated vapor pressure were obtained from the Chemspider website (http://www.chemspider.com/ accessed on 18 November 2022), which uses the Modified Grain method to provide calculated vapor pressure values in mm of Hg [30].

### 2.10. Statistical Analysis

Data related to antimicrobial and antifungal activities were expressed as the standard error of the mean (±SEM). For optical density measurements, data comparisons were performed using one-way analysis of variance (ANOVA), and post hoc analysis was carried out by the Student Newman–Keuls (SNK) test. GraphPad Prism (Version 5.03, GraphPad Software, San Diego, CA, USA) was employed for data analysis and generation of charts. Statistical significance was set at * *p* < 0.05, ** *p* < 0.01, and *** *p* < 0.001.

## 3. Results and Discussion

The selenocyanates selected for this study exhibited significant antimicrobial activities against each of the bacteria and fungi tested. Notably, thiocyanate and isothiocyanate were both virtually inactive in these assays. Equally notably, the activity of the selenocyanates could also be observed via gas phase interactions, and this was despite the comparably low(er) vapor pressure of the selenium versus sulfur analogues. These findings are now presented and discussed in more detail.

### 3.1. Antibacterial Activity

**Figure 2:**
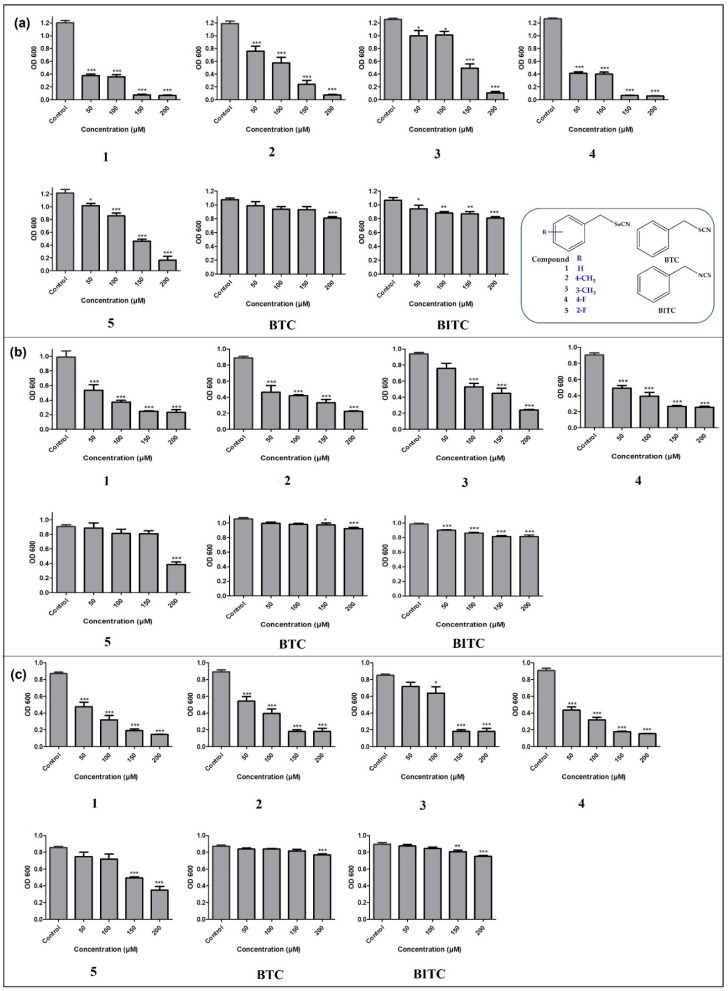
Impact of selenocyanates and thiocyanates on the growth of *E. coli* (Panel (**a**)), *P. syringae* (Panel (**b**)), and *M. luteus* (Panel (**c**)). The insert in Panel a represents the chemical structures of compounds employed in this study. Values represent mean ± S.D. * *p* < 0.05, ** *p* < 0.01 and *** *p* < 0.001. See text for further experimental details.

Gram-negative bacteria currently present a particular challenge in the development of effective antibiotics. Whilst the sulfur-containing compounds (BITC and BTC) have a relatively low activity against *E. coli* (Figure 2, Panel a), inhibition of bacterial density is much more pronounced in the case of selenium compounds, even at the lowest concentrations employed (50 µM). Compounds 1 and 3 have manifested strong antibacterial activity against *E. coli*, inhibiting the bacterial growth greater than 60% at a concentration of 50 µM. Similarly, compound 2 has also inhibited the bacteria in a concentration-dependent manner. Compounds 1–4 have almost completely inhibited the growth of *E. coli* at the highest concentration employed, i.e., at 200 µM. Intriguingly, compound 4, which contains an electron withdrawing fluorine atom at the *para* position, has provided better antimicrobial activity as compared to its analogous compound 5 with fluorine at *ortho* position. Moreover, compound 4 has provided better anti-bacterial activity against *E. coli* when compared to compounds 2 and 3 containing electron-donating methyl group at *para* and *meta* positions, respectively (Figure 2, Panel a). Compound 2 has provided better anti-bacterial activity against *E. coli* when compared to compound 3 (Figure 2, Panel a).

A similar picture also emerges in the case of *P. syringae*. The inhibition of bacteria by the sulfur-containing compounds was statistically significant yet minor compared to their selenium-containing analogues, which, at a concentration of 200 µM, inhibited bacterial growth by 80%, whereas BTC and BITC at these concentrations achieved only 20% inhibition (Figure 2, Panel b). Interestingly, the wild-type strain *P. syringae* pv. tomato DC3000 was rather resistant towards even the most aggressive sulfur compounds, such as aliphatic isothiocyanates (sulforaphane), thanks to specific resistance genes (so-called *sax* genes) which mediate protection [31,32]. The *sax* genes are primarily required to annihilate isothiocyanate-based defenses, which accelerates a disease outcome, particularly in the young leaves crucial for plant survival [31,32]. It now looks as if these genes do not protect against the reactivity of the selenium compounds. Compounds 1–4 inhibited the growth of *E. coli* by almost 80% at the highest concentration of 200 µM. Compounds 1, 2, and 4 inhibited the growth of *P. syringae* by around 50% at the lowest concentration of 50 µM (Figure 2, Panel b).

Figure 2 Panel c summarizes the activity of selenocyanates and thiocyanates against Gram-positive bacterium *M. luteus*. The antibacterial activity of seleno-compounds was similar to that observed in case of Gram-negative bacteria. Here, again, compounds 1–4 exhibited concentration-dependent growth inhibition of *M. luteus*, whilst the sulfur compounds BTC and BITC were more or less inactive. Compounds 1, 2, and 4 inhibited the growth of bacteria, even at the lowest concentration of 50 µM (Figure 2, Panel c).

The results obtained in the quantitative liquid media culture studies were more or less confirmed by two additional, independent series of tests, namely the drop test method and the Petri dish diffusion method. In essence, they mirrored the findings in liquid media, whereby the selenium compounds 1–5 showed a pronounced, concentration-dependent toxicity against the bacteria, whereas the sulfur analogues BTC and BITC were as good as inactive.

Notably, compounds such as 2 and 4 were more active in the drop test and completely inhibited the growth of bacteria in medium containing the highest bacterial content (10^−1^ dilution), whilst the sulfur compounds did not affect the growth of bacteria in medium containing the least amount of bacteria (10^−6^ dilution) at a concentration of 100 µM. The results of drop test also indicate that selenocyanates exhibited slightly higher toxicity against Gram-positive bacteria when compared to Gram-negative bacteria.

A similar trend has been affirmed by the plate diffusion test, which revealed that selenocyanates, in general, have provided zones of inhibition even at the lowest concentration of 2 mM, whilst BTC and BITC provided no zones of inhibition even at the highest concentration of 8 mM. Moreover, compounds 1–5 also produced greater inhibition zones in case of *M. luteus* (Gram-positive) as compared to *P. syringae* (Gram-negative).

### 3.2. Antibacterial Activity via the Gas Phase Assay

In the current investigation, diverse test procedures have been chosen for the determination of antimicrobial activity, since bacterial growth in a liquid culture is different from the growth in colony on a plate or embedded in an agar medium, due to, for instance, a much slower diffusion in agar compared to liquid media. Similarly, bacteria under the influence of compounds behave differently in the gas phase assay. This variance leads to altered susceptibility of different microorganisms to target compounds, and also to the question of whether these selenium compounds, perhaps similar to sulfur compounds such as allicin, may also react “at a distance”, via the gas phase [26,33,34].

**Figure 3:**
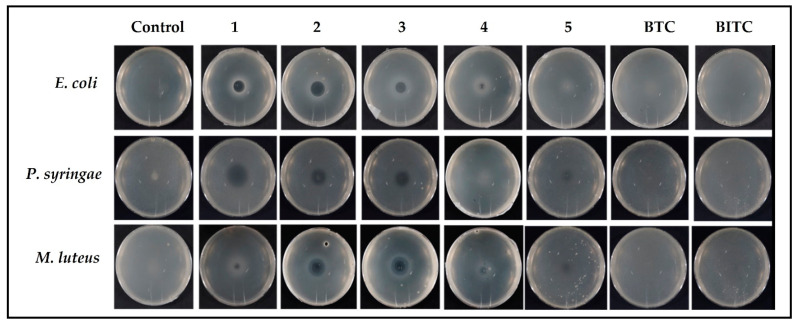
Selenocyanates provide antimicrobial activity against *E. coli*, *P. syringae*, and *M. luteus* via the gas phase, whilst thiocyanates (BTC and BITC) remain totally ineffective against the bacteria selected.

As part of the relevant gas phase assay, a 20 µL drop of 80 mM test solution of compound was placed in the center of a Petri dish lid and its base, containing agar medium seeded with bacteria, was inverted above the lid [33]. Hence, compounds diffused through the air inside the growth medium containing bacteria, and there was no direct contact between the agar and compound under investigation. Despite the fact that compounds 1–5 are solids and, hence, not supposed to be particularly volatile, with calculated vapor pressures ranging from 0.000349 mm of Hg to 0.00117 mm of Hg at 25 °C for 2 and 4, respectively, compounds 1–3 produced an impressive inhibition zone above the Petri dish lid for *E. coli*, *P. syringae* and *M. luteus*, as visible in Figure 3 [35,36,37]. Compound 4 was less active, resulting in a small zone of inhibition at the center of the Petri dish. The sulfur containing BTC and BITC, despite 50-fold higher calculated vapor pressures of 0.0457 mm of Hg and 0.0355 mm of Hg at 25 °C as compared to compound 1, with calculated vapor pressure of 0.000908 mm of Hg at 25 °C, respectively, were inactive under those conditions [35,38,39]. Allyl isothiocyanate (AITC), a very closely related sulfur-based fumigant, has a vapor pressure of 3.7 mm of Hg. AITC been reported to inhibit the growth of *E. coli* with an IC_50_ value of 34 ng mL^−1^(0.34 µM) in gas phase [18]. This remarkable difference of antimicrobial activity in gas phase can be associated not only with the difference in the vapor pressure, but also to the different chemical structures of these molecules. These findings are rather intriguing, as they endow such organic selenocyanates with a “remote” mode of (cyto-)toxic action which, depending on selectivity, may be rather useful in the context of fumigation and attacks against airborne microbial organisms [40].

### 3.3. Determination of Zone of Fungal Growth

**Figure 4:**
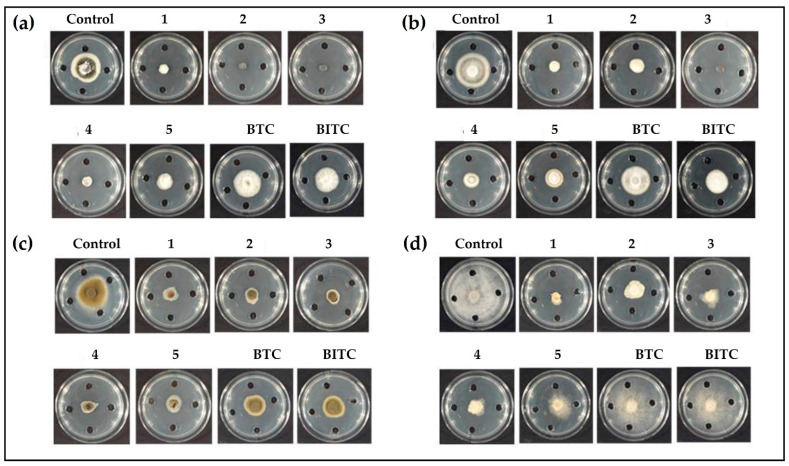
Impact of selenocyanates and thiocyanates on the growth of (**a**) *V. dahliae*, (**b**) *V. longisporum*, (**c**) *A. brassicicola* and (**d**) *B. cinerea*.

Besides bacteria, various unicellular fungi are also prominent targets of selenium compounds [14]. Thus, the antifungal activity of compounds has been determined at a concentration of 4 mM in assays involving fungal plant pathogens, including *V. dahliae*, *V. longisporum*, *A. brassicicola*, and *B. cinerea*. A significant decrease in the growth of fungi has been observed in plates treated with the organic selenocyanates when compared to the control plates. Compounds 1–5 have resulted in significant growth reduction of *V. dahliae*. Compounds 2 and 3 (laced with electron-donating methyl group at *para* and *meta* positions, respectively) inhibited growth entirely, followed by compounds 1 and 4. The least inhibitory effect, amongst selenocyanates, has been observed for compound 5 (Figure 4, Panel a). The sulfur compounds BTC and BITC have been observed to be ineffective against *V. dahliae* (Figure 4, Panel a). In case of *V. longisporum*, compound 3 is once again the most active one by completely inhibiting fungal growth. Compound 1 has also significantly inhibited the growth of *V. longisporum*, followed by 2 and 4, with the least activity observed for 5 (Figure 4, Panel b). Here again, both sulfur compounds have not been very effective in inhibiting the growth of *V. longisporum*. BITC has provided better inhibitory action when compared to BTC (Figure 4, Panel b). In the case of *A. brassicicola*, compounds 1–5 have almost completely constrained the fungal growth. Intriguingly, BTC and BITC have also provided some inhibitory effect, although not as strong as selenocyanates. (Figure 4, Panel c). Selenocyanates 1–5 have also provided excellent antimicrobial activity against *B. cinerea*. Here, compound 1 has provided the maximum inhibitory effect, followed by 3 and 4 (Figure 4, Panel d). Interestingly, the thiocyanates, in particular BITC, also decreased the growth of fungi under investigation, such as *V. dhaliae*, *V. longisporum*, and *A. brassicicola*, although the activity was lower in comparison to the selenocyanates. *B. cinerea*, however, exhibited resistance to thiocyanate and isothiocyanate treatment, and there was no considerable difference in the control and thiocyanate-treated plates. The lower antifungal of BTC is rather surprising, as the same compound has been reported to inhibit the growth of other fungi, including *Candida albicans* and *Neurospora crassa*, with very low IC_50_ values of 0.1 mg L^−1^ and 0.01 mg L^−1^, respectively [41,42].

**Figure 5:**
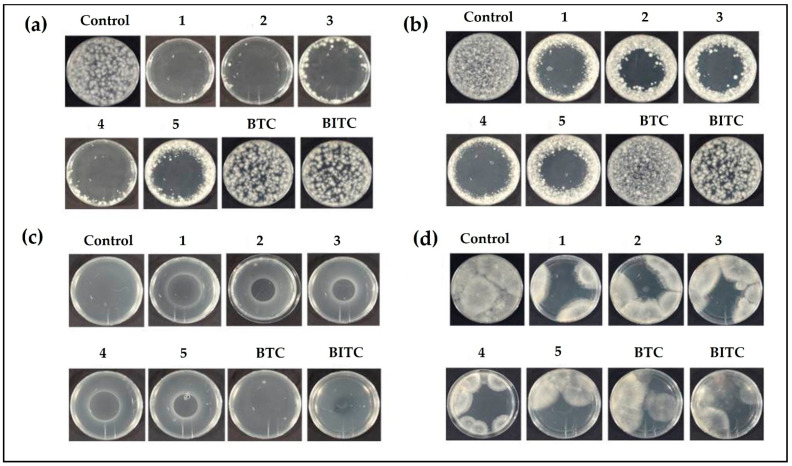
Antifungal activities of selenocyanates and thiocyanates via the gas phase against (**a**) *V. dahliae* (**b**) *V. longisporum* (**c**) *A. brassicicola* and (**d**) *B. cinerea*.

Antifungal activity of 1–5 and thiocyanates in the gas phase has been also determined, and, rather surprisingly, clear zones of inhibition with fairly sharp borders have been observed (Figure 5). This emergence of sharp borders can be interpreted as the concentration gradient of compound diffusing away from the central drop into the air in the closed Petri dish, and a tight concentration threshold for inhibition of fungi in the agar medium. In the case of active compounds, an inhibition zone is visible in agar medium above the droplet in the Petri dish lid. Interestingly, compounds 1–4 have almost completely inhibited the growth of *V. dahliae*. Similarly, compound 5 has restricted the growth of *V. dahliae* to the boundary of the Petri plate (Figure 5, panel a). A similar yet slightly lesser antimicrobial trend has also been observed for compounds 1–5 against *V. longisporum* (Figure 5, Panel b) and *A. brassicicola* (Figure 5, Panel c). Unlike the aforementioned fungi, *B. cinerea* provided a certain resistance towards each of the compound, as no clear and sharp boundaries were observed (Figure 5, Panel d). The inhibition of fungal growth, however, suggests that selenocyanates 1–5 provide anti-fungal activity against *B. cinerea*. The thiocyanates did not inhibit the growth of *V. dahliae* and *V. longisporum*. BITC provided some inhibitory activity against *B. cinerea* and a very minute inhibitory activity against *A. brassicicola*. BTC was ineffective against all the fungi except *B. cinerea*, where a slight inhibition has been observed. The antifungal activities of the organic selenocyanates have reflected the antibacterial activities observed in the bacterial assays, affirming a generally low activity associated with the corresponding organic thiocyanates. AITC inhibited the growth of *V. dahliae*, with an EC_50_ value of 0.264 mg L^−1^, and *A. brassicicola*, with an IC_50_ value of 2.9 mM [43,44]. Moreover, AITC inhibited both the mycelium and conidia of *B. cinerea* with EC_50_ values of 1.35 mg L^−1^ and 0.62 mg L^−1^, respectively [45]. The volatile glucosinolates from the leaves of *Arabidopsis thaliana* have also been reported to inhibit the growth of *V. longisporum* [46].

## 4. Conclusions

In summary, the organic selenocyanates exhibit reasonable antibacterial activities against pathogenic bacteria and plant pathogenic fungi. The efficacy of selenocyanates against bacteria and fungi is far superior compared to the thiocyanates. Moreover, activity of the organic selenocyanates by diffusion through the gas phase may suggest a potential application of these compounds in the context of fumigation or even for the treatment of lung diseases, since conventional antibiotics are generally not volatile. Furthermore, applications of these agents in agriculture—for instance, fumigation of soils, treatment of crops against phytopathogens, and protection of seeds against seed-borne diseases—may be considered.

Future studies are required to focus on the question of selectivity, especially at low(er) concentrations of these compounds, cellular targets, mode(s) of action, and, of course, stability and metabolism in more complex *in vivo* systems, including the human organism.

## Data Availability

Not applicable.
